# Photoperiodic Programming of the SCN and Its Role in Photoperiodic Output

**DOI:** 10.1155/2018/8217345

**Published:** 2018-01-09

**Authors:** Michael C. Tackenberg, Douglas G. McMahon

**Affiliations:** ^1^Vanderbilt Brain Institute, Vanderbilt University, Nashville, TN, USA; ^2^Department of Biological Sciences, Vanderbilt University, Nashville, TN, USA

## Abstract

Though the seasonal response of organisms to changing day lengths is a phenomenon that has been scientifically reported for nearly a century, significant questions remain about how photoperiod is encoded and effected neurobiologically. In mammals, early work identified the master circadian clock, the suprachiasmatic nuclei (SCN), as a tentative encoder of photoperiodic information. Here, we provide an overview of research on the SCN as a coordinator of photoperiodic responses, the intercellular coupling changes that accompany that coordination, as well as the SCN's role in a putative brain network controlling photoperiodic input and output. Lastly, we discuss the importance of photoperiodic research in the context of tangible benefits to human health that have been realized through this research as well as challenges that remain.

## 1. Introduction

Outside of the tropics (where day length remains relatively consistent throughout the year), the changing photoperiod is a reliable and predictable seasonal signal that presents an opportunity for organisms to adapt to seasonal changes in factors such as temperature and resource availability in an anticipatory fashion. The first scientific observation that changing day length was a critical factor in seasonal responses was reported in 1920 by Garner and Allard [1]. In that study, they observed that certain plants could only attain the flowering and fruiting stages of their development when housed in particular day lengths: while some species required long days and others required short days, those light durations were the critical factors in their reproductive cycle. Since that time, seasonal responses have been described and studied in a wide variety of different organisms [2–5]. More recently, seasonal factors have been linked to human health. While we have benefitted greatly from understanding the seasonal responses of the organisms on which we rely for survival, evidence is emerging that we are also photoperiodic organisms ourselves [6]. Over the last several years, much research has been done on the relationship among photoperiods, especially extreme photoperiods, and psychiatric health, sleep, and noncommunicable diseases [7–12].

The ability of organisms to exhibit daily biological timekeeping is well-established. Organisms ranging from bacteria to humans have innate, endogenous 24-hour biological oscillations that persist into constant conditions and can be entrained by the external light-dark cycle of the Earth. These rhythms allow for a myriad of time-dependent functions, including predator avoidance [13], sun-based navigation [14–16], metabolic regulation during hibernation and torpor [17–20], and photosynthetic efficiency [21–23]. In mammals, these daily rhythms are orchestrated by a central circadian pacemaker located in the hypothalamus, the suprachiasmatic nucleus (SCN) [24]. The SCN consists of approximately 20,000 neurons and is not a homogenous structure. It can be divided into two major subregions based on neurochemical composition, connectivity, and spatial position. These subregions are typically referred to as the dorsomedial (also referred to as the dorsal or shell SCN) and ventrolateral (ventral or core SCN). The SCN receives input from the retina directed towards its ventrolateral subregion, while its dorsomedial region features numerous direct and indirect projections to other hypothalamic nuclei controlling homeostatic function and to the rest of the brain [25]. When considering the SCN's primary function to integrate external light information with endogenous timekeeping and then to orchestrate a wide range of behavioral and physiological responses, the structure is a prime candidate for controlling seasonal rhythms.

The first suggestion that circadian pacemakers were involved in orchestrating seasonal responses was made by Bünning in 1936 [26] and refined in 1960 [27]. He proposed that a portion of a plant's circadian cycle was light-requiring, or “photophilic,” while the other portion was dark-requiring, or “scotophilic.” In this model, when a short-day plant in its photophilic phase is exposed to dark, a short-day mechanism is successfully initiated, while a long-day plant in its scotophilic phase exposed to light will have a long-day mechanism successfully induced. This theory was accepted and further refined by Pittendrigh and Minis, who suggested that it overlooked the primary function of the circadian pacemaker's response to light to set the phase of the pacemaker [28]. This combined view, in which the master circadian pacemaker integrates information about both the time of day and the length of day, was a critical factor in the way Pittendrigh viewed the circadian clock and its entrainment by light, for the following thirty years [29]. The experimentation of Pittendrigh et al. on entrainment theory resulted in an increased understanding how circadian pacemakers entrain, or synchronize, to the light cycle. Notably, how a circadian rhythm synchronizes to a light-dark cycle to produce stable entrainment is dependent on the length of the photoperiod, with different photoperiods producing characteristic phase angles of entrainment [28].

When the SCN was conclusively identified as the locus of a mammalian master clock, investigations began into the mechanisms of mammalian circadian entrainment at a cellular level. In 1995, it was established that the duration of the light-sensitive phase of the SCN, measured via *c-fos* gene expression, was longer in rats housed in short photoperiods than those housed in long photoperiods [30]. When rats were switched from long to short photoperiod, the light responsive phase took as long as two weeks to reach the decompressed short-photoperiod phenotype, demonstrating that there were lasting effects of photoperiod within the SCN [31].

Later, invaluable descriptions of the cellular and network properties of the SCN began to emerge and continue through today (for review, see [32]). These findings enabled new approaches in determining the biological mechanism for mammalian photoperiodic encoding [33, 34], as well as in elucidating the neurophysiological basis for seasonal outputs like depression-anxiety behavior and reproduction. A principal goal of this line of research, which continues today, is to define the neurobiological network that orchestrates photoperiodic responses. While future improvements to our understanding of this network will produce benefits for medicine and society, current research on the subject has already yielded useful applications in clinics and beyond.

## 2. The SCN as a Site of Photoperiodic Encoding

Though the idea that the circadian pacemaker can encode seasonal information was generally accepted by the circadian field in the 1960s, at that time, the mechanistic basis of seasonal encoding was difficult to examine with the experimental tools at hand. Substantial progress on the understanding of the cellular and molecular mechanisms underlying daily oscillations in the SCN provided a greatly enhanced basis to study the mechanisms of encoding the season light cycle. A key piece of the puzzle was the elucidation of the molecular mechanisms of the mammalian circadian clock-works. This provided not only critical insight into the molecular and genetic foundations of daily timekeeping, but in addition, it provided a whole new generation of experimental tools. Since the clockwork was found to be a gene-based oscillator, probes and transgenes that enabled real-time gene expression analysis provided a new way to visualize and quantify circadian rhythmicity in SCN neurons and networks.

Within each SCN neuron, a genetic feedback loop known as the transcription-translation feedback loop (TTFL) was found to be the foundation of the autonomous near 24-hour rhythms in gene expression and neuronal firing rate (for reference, see [35]). In this loop, the transcription of the *Period* (*Per1* and *Per2*) and *Cryptochrome* (*Cry1* and *Cry2*) genes is initiated through the CLOCK∷BMAL1 transcription factor complex acting on E-box enhancer elements in the promoter regions of *Per* and *Cry* (and many other circadian-regulated genes). The protein products of the *Per* and *Cry* genes then accumulate in the cytoplasm, where they heterodimerize and reenter the nucleus to inhibit their own transcription. This process takes roughly 24 hours to complete, thus forming a self-sustaining circadian oscillation in both core clock genes and other circadian-regulated genes. This feedback loop contributes to intracellular calcium rhythms and rhythmic electrical activity through daily oscillations in ion channel and receptor transcription [32]. The result of these interlocking rhythms is a particular daily waveform of circadian gene expression and firing rate within individual SCN neurons and across the SCN neuronal network, in which the degree of synchrony between individual SCN neuron gene expression or electrical activity cycle sets the overall waveform of SCN gene expression or electrophysiological activity [36–38]. These waveforms have been extensively studied in equinox (12 hours light : 12 hours dark) conditions. Generally, SCN neurons fire at a higher rate (approximately 6–10 Hz) during the day and at a lower rate (approximately 1-2 Hz) during the night. This firing pattern persists into constant conditions. Light pulses during the low-firing phase at night have been shown to transiently increase firing rate and gene expression as part of a phase-shifting response of the clock [39–41].

Do the cellular or network gene and electrical rhythm waveforms change across different photoperiods? If so, this change could form a basis for photoperiod encoding. Reports from studies on hamsters [42], mice [43], and sheep [44] showed *Per1* mRNA levels had a longer elevated phase when assayed during long days compared to short days. This result, however, could be explained by the light activation from the retina increasing neuronal activity and, as a result, *Per1* transcription. Sumová and colleagues addressed that possibility by assaying the *Per1* (as well as other clock gene) mRNA levels in the SCN of rats that had been housed in different photoperiods but were then transferred to constant darkness, thus eliminating the proximal effect of light exposure [45, 46]. In these tests, the high-expression phase of the *Per1* mRNA rhythm was found to lengthen in rats previously housed in long days when compared to short days. *Cry1* mRNA was also found to have significant differences between long- and short-photoperiod rats, but *Bmal1* and *Clock* mRNA were not significantly affected by photoperiod.

When asking a similar question about the effect of photoperiod on electrical activity within the SCN, Schaap and colleagues found that different photoperiods altered the waveform of the spike frequency rhythm of the SCN, extending the high-firing phase and contracting the low-firing phase in long days, with the opposite effect occurring in short days [47]. Simulations suggested that while SCN neurons fire individually in the same waveform regardless of photoperiod (i.e., each individual neuron's high-firing phase duration remains unchanged across different photoperiods), changing their phase relationship can combine to produce the firing rate waveform changes observed between the two photoperiods. Further investigation into the phase distribution of SCN neurons in long or short photoperiod revealed that while single SCN neurons do in fact maintain the same daily firing rate profile across short and long photoperiods, the phase relationship between the neurons does in fact change, broadening in long photoperiods [48]. Interestingly, a population of approximately 50 neurons is required before a differentiable photoperiodic profile emerges. Thus, a key finding of these studies was that photoperiod was encoded in the SCN at the network level, rather than at the cellular level.

Studies examining the photoperiodic response of gene expression rhythms within the SCN have found results similar to those detailing the response of rhythms in firing rate. Inagaki reported that while individual neurons maintained the same waveform of *Per1* transcriptional activity read out with *Per1∷luc* expression across different photoperiods, their phase relationships to one another were more widely distributed in long photoperiods than in short [49]. Ciarleglio et al. found similar results when examining the effect of proximal photoperiods on the *Per1* transcriptional activity rhythm read out by a *Per1∷GFP* reporter [50]. Thus, increasing photoperiod (long days) was found to increase the phase dispersal of both single SCN neuron gene and electrical activity. Interestingly, fitting with this trend, constant light, which can be considered the most extreme long photoperiod, can result in a complete dispersal of SCN neuron phasing, producing both behavioral and SCN tissue level arrhythmicity while essentially randomizing the phase of the still cycling individual SCN neurons [51]. Clear parallels exist between the rhythmic profiles of firing rate and circadian gene expression in the SCN across photoperiods, demonstrating that the photoperiodic response of the SCN goes beyond simple light-driven changes, including a persistent change at the network level. These results, in conjunction with the spike frequency rhythm waveform studies already discussed, provide ample evidence that phase variance of SCN neurons is an encoding mechanism for photoperiod within the SCN.

The results described above were obtained by exposing adult rodents to different photoperiods. What, then, are the effects of photoperiods experienced during development? In terms of the development of clock gene rhythms in the SCN, the effect of photoperiods on the timing and waveform of these rhythms was shown to be measurable by P10 in the rat [52] suggesting that the interval photoperiod encoding begins during the perinatal period. In addition, Cambras and colleagues showed in a series of studies that noncircadian lighting conditions (principally LL and DD) experienced by rodents during development and maturation can have lasting effects on the circadian system and its responsiveness light [53–56]. Ciarleglio et al. subsequently studied the impact of entrainment to different photoperiods during perinatal development on the resulting cellular and network properties of the SCN and on locomotor behavior rhythms [50], asking whether exposure to different seasonal photoperiods during development and maturation can have enduring effects on clock properties. Interestingly, in contrast to photoperiod encoding in adult animals that acts primarily at the network level altering the phase distribution of clock neurons, perinatal photoperiods induced enduring changes in the waveform of single SCN neurons to program overall SCN gene expression waveform later in life. Consonant changes were apparent in locomotor behavioral rhythms and in the phase stability of SCN entrainment to different photoperiods in adulthood. Thus, results to date suggest that while photoperiod encoding primarily resides at the network level in the mature SCN (but see [57]), during development, photoperiod programs rhythms at the cellular level in a manner that persists into adulthood.

As mentioned previously, the SCN is a heterogeneous structure, with two principal regions identified by their neuropeptide gene expression—the ventrolateral region by vasoactive intestinal peptide (VIP) expression and the dorsomedial region by arginine vasopressin (AVP) expression. Are different SCN regions differentially involved in encoding? In terms of rhythmicity and acute responses to light, the predominantly retinorecipient ventrolateral SCN has been shown to lag behind the dorsomedial SCN in gene expression ([58], but see [36]) by approximately 1 hour but has also been shown to respond to phase-shifting light stimuli rapidly while the dorsomedial SCN shifts more slowly [40, 59–61]. Because of this steady phase angle difference, as well as photoperiod-dependent differences in c-Fos rhythms in the two regions [62], differences between the phasing of the two regions of the SCN between short and long photoperiods became an experimental focus. Brown and Piggins found that the phasing of the electrical activity in the dorsal SCN was less tightly organized than the ventral SCN in slices collected from animals in equinox conditions and that the electrical activity profile of the dorsal SCN did not change across short or long photoperiods. The overall SCN waveform change in electrical activity observed in long days versus short days was found to be caused by changes within the ventral SCN alone [63]. Likewise, AVP mRNA profiles have been shown to be altered between long and short photoperiods, indicating a change in profile within the dorsomedial SCN [64]. Significant phase differences have also been demonstrated between the rostral/anterior and caudal/posterior SCN [65]. Those differences are enhanced in long photoperiods compared to short [66–68]. Buijink and colleagues found that phase distribution differences in PER2 expression under long day conditions were associated with changes in period stability within the dorsolateral anterior SCN [69]. Looking at the SCN along the anterior-posterior axis rather than the dorsal-ventral axis, using coronal slices, both Inagaki using *Per1∷luc* [49] and Buijink using PER2∷LUC [69] found that the photoperiodic differences in the phase variance of cellular gene expression rhythm profiles were emphasized in the anterior SCN versus the posterior. The functional significance of the regional differences in phase distribution remains poorly defined. One possibility is that subregions with large phase differences comprise the morning and evening (M and E) oscillators proposed by Pittendrigh and Daan [70]. Inagaki and colleagues observed a bimodal distribution of the phase of anterior SCN neurons in long photoperiods, corresponding to these M and E oscillators [49]. Recent work by Yoshikawa and colleagues used horizontal slices, as opposed to the previously-used coronal slices, to better localize putative M and E oscillators along the anterior-posterior axis [71].

Considering that the dorsal SCN is the source of many of SCN efferents, a question arises about how seasonal information encoded within the phase distribution of ventrolateral SCN neurons is relayed to the dorsomedial SCN for signaling to the rest of the brain. Neurotransmission within the SCN is a major research focus, with many signaling molecules released within the SCN. The phase difference between the dorsal and ventral SCN has been shown to increase as photoperiod length increases, with resynchronization between the two regions after transfer to constant conditions being sensitive to TTX, GABA_A_ blockers, and VIP antagonists [72]. GABA, the predominant neurotransmitter within the SCN, was found to have a significant role in the coupling of dorsomedial and ventrolateral SCN, with the addition of the GABA_A_ blocker bicuculline to SCN slice cultures mimicking the effect of physically disconnecting the two SCN subregions with a knife cut [73]. In that study, Albus and colleagues found that the transient response to a phase-shifting light pulse involved a bimodal distribution of electrical activity during the phase resetting. When the SCN was cut in half, separating dorsomedial from ventrolateral SCN, the electrical response to the light pulse was unimodal for each region, but the two regions were out of phase. The retinorecipient ventrolateral SCN half was more responsive to the light pulse, with the dorsomedial SCN not undergoing a significant shift. Interestingly, the blockade of GABA_A_ receptors with bicuculline yielded similar results to the physical cut, with unimodal electrical activity in each half and a significantly smaller phase shift within the dorsomedial region as compared to the ventral region. This study provided compelling evidence that GABA_A_ receptors mediate dorsal-ventral SCN coupling. Later, when the spatial relationship of the photoperiodic response within the SCN became clearer, the GABA-mediated signaling results took on new meaning. Farajnia and colleagues followed up on this line of research in 2014, showing that the dual role of GABA within the SCN (acting as both an excitatory and inhibitory neurotransmitter) may be an integral part of the SCN's photoperiodic response [74]. In that study, excitatory GABAergic activity was found to be higher during the day in a long photoperiod than at night, while in a short photoperiod, excitatory GABAergic activity was found to be lower during the day than at night. Closer examination of the electrical properties of GABA signaling in these two photoperiodic conditions revealed a differential rate of excitatory and inhibitory currents. The period instability observed by Buijink and colleagues within the anterior SCN during long photoperiods can contribute to the SCN reaching the altered phase distribution among its constituent neurons [69]. This period-instability mechanism may occur in conjunction with active decoupling that was demonstrated in models described by Myung and colleagues, in which differential effects of GABA reception are used as attractive and repulsive forces within the SCN network [75].

The phase distribution of SCN neurons found to be a characteristic of a particular photoperiod is primarily driven by neurons in the ventral SCN, which is often defined by its expression of VIP, an important neuropeptide for interneuronal coupling in the clock. VIP was found to be required for the persistence of the photoperiodic electrical activity profile of the SCN into constant conditions [76]. This result suggests that the VIPergic population of the SCN, mostly within the ventral half, is necessary for encoding at least the duration of locomotor activity.

## 3. The SCN and Photoperiodic Response Signaling through Melatonin

How then does the SCN fit into a putative photoperiod response network in the brain? An important aspect of creating a map that links light input to the retina with photoperiodic outputs is the understanding of the direct neurophysiological basis of each of those outputs. Once these neural correlates are understood, a framework can be made for how differing durations of light exert effects on those structures. While in many cases these brain structures have been defined, the enumeration of each of these cases is beyond the scope of this review. Rather, we will focus on examples of those structures and outputs that may be directly related to the SCN's photoperiodic role, particularly in conjunction with the SCN's indirect projections to the pineal gland and its role in regulating melatonin rhythms.

The release of melatonin by the pineal gland is a prime candidate for transmitting photoperiodic cues throughout the brain (for review, see [77, 78]). Melatonin is clearly involved in seasonal control of reproductive behavior, one of the most well-studied photoperiodic outputs [79]. Seasonal reproductive behaviors have been shown to be sensitive to melatonin applications and are ablated with removal of the pineal gland, the source of melatonin in the brain [80–82]. Further upstream, those seasonal changes were found to be mediated by the periventricular nucleus (PVN) and the SCN [83, 84]. This forms a photoperiodic response circuit, with the SCN projecting indirectly to the pineal gland through a chain of polysynaptic connections that include the PVN. In addition to reproductive behavior, body weight changes associated with different photoperiods (which have been shown to be influenced by melatonin, [85]) have been traced to hormone and neuropeptide signaling differences within the arcuate nucleus of the hypothalamus [86]. Interestingly, while long infusions of melatonin replicate short days in Siberian hamsters and induce a reduced body weight phenotype, SCN lesions eliminate those effects, suggesting a role for the SCN both downstream and upstream of melatonin signaling in the photoperiodic signaling network [80]. Another target for indirect melatonin signaling from the pineal is the Raphe nucleus. Recent evidence shows that changes in Raphe serotonin neuron firing rate across different developmental and proximal photoperiods are melatonin-dependent, with melatonin-receptor knockout mice lacking photoperiod-specific firing rates [87].

Not all photoperiodic effects are melatonin-dependent, however. Recent work on C57BL/6 mice, which do not produce substantial amounts of melatonin, has shown that mice in long photoperiods undergo body weight increases and have altered plasma metabolic profiles [88]. Earlier work in the same mouse model and in Fischer 344 rats shows an altered corticosterone profile in long versus short days [89]. Mice with genetic defects in melatonin synthesis have been shown to have modulated locomotor behavior in different photoperiods, manifested as shorter durations of activity in long photoperiods and longer durations of activity in short photoperiods [90]. The work discussed previously demonstrating the necessity of VIP neurons in the persistence of long and short-day locomotor behavior duration patterns into constant conditions was also performed in the melatonin-deficient 129/Sv mice, demonstrating that the persistence of this photoperiodic output is not melatonin-dependent [76]. With few afferent connections, the retina may be an example of an SCN- and melatonin-independent photoperiodic system, with different day lengths altering ERG responses in a persistent manner [91].

In the retina and Raphe, as in the SCN itself, photoperiods experienced during the development and maturation of these neural circuits have been demonstrated to have enduring effects that shape sensory perception and behavior in adulthood. Within a circadian context, developmental photoperiod was shown to persistently change the waveform of circadian *Per1* promoter activation in the SCN, as read out by *Per1∷GFP*, as well as the phase angle of the peak relative to dusk and the duration of the locomotor activity phase [50]. Photoperiod effects within the mouse retina are development-dependent, with animals raised on short photoperiods continuing to have reduced retinal sensitivity (short-photoperiod ERG phenotype) despite being moved to long photoperiods during adulthood [91]. In the Raphe nucleus, enduring changes in serotonin neuron firing rate were found to be dependent on the developmental photoperiod, with proximal photoperiod playing a less prominent role [87]. Mice matured in long, summer-like photoperiods exhibit increased serotonin neuron firing, increased midbrain serotonin content, and decreased depression and anxiety-like behaviors in adulthood. In the extreme of constant light, the disruption of the SCN and behavioral rhythms observed in adult mice [51] is much more dramatic and pervasive with perinatal exposure [92]. The SCN is likely to play a role in the encoding of lasting developmental photoperiod effects, with recent work demonstrating that individual SCN neurons exhibit enduringly altered *Per1::GFP* expression rhythm profiles across different developmental photoperiods [50]. In contrast to studies mentioned above where gene expression and firing rate rhythms remained consistent across photoperiods but changed their phase relationship in adult animals exposed to different photoperiod, this work focused on animals reared in particular photoperiods. Photoperiods experienced during the perinatal period had lasting effects into adulthood on the gene expression waveforms of individual SCN neurons, their phase relationships to the light cycle, and their intrinsic periods [50]. Considering the influence that the SCN has on the rest of the brain, enduring changes within the SCN during development may help explain the persistent effects of early-development photoperiod observed in other tissues, such as the Raphe. The precise timing of this critical developmental window, as well as the underlying changes within the SCN neurons that lead to the altered neuronal period, remains undetermined.

It remains an open question which photoperiod-dependent behaviors and physiological responses are mediated through the SCN, regardless of how well the photoperiod may imprint onto the phase distribution of the SCN neurons. While the inputs and outputs of the SCN are a consistent subject of study, most of these connections are examined in the context of circadian timing and regulation. Moving forward, careful experimentation will be required to determine the role that the SCN, or any other structures capable of encoding seasonal light information, plays in a particular photoperiodic output. Further, these studies will need to differentiate between those photoperiodic effects that are directly driven by light, those that persist for some length of time even after photoperiodic input has ceased, and those that are imprinted developmentally and produce lasting changes to physiology.

## 4. Applications in Medicine and Society

Whereas the evidence for seasonality driven by photoperiodism in many animals and plant species is widespread and compelling, such evidence is less developed for humans. Still, there are dramatic examples of the impact of seasonality on human health, particularly in the developmental domain, consonant with the developmental origins of adult disease. There is also demonstrated promise of the application of circadian photobiology to address these issues. For example, in 2015, Boland et al. reported the results of a “SeaWAS” (Season-Wide Association Study) in which they analyzed the medical records of more than 1.7 million patients at Columbia University Medical Center for associations of medical diagnoses for 1688 diseases with season of birth [93]. They found significant associations with 55 diseases including ADHD, asthma, and 9 cardiovascular conditions. This approach, combined with PheWAS analysis of genomic data, holds great promise in revealing the gene by environment interactions underlying disease risk from developmental exposures to season variables.

Therapeutic intervention based on circadian photobiology has already begun in the clinic. Circadian light therapy has long been recognized as a successful treatment for seasonal depression [94]. Another salient recent example is the work of Ohta and colleagues who defined perinatal exposure to constant light as a potential risk factor for disruption of the developing mammalian biological clock in mice [92] and then applied this to the neonatal intensive care unit (NICU) where infants may be exposed to constant light. To ensure proper circadian light cycles for premature infants and the ability of medical staff to provide continuous care, Ohta and colleagues targeted stabilization of the developing clock by selective optical filtering of wavelengths that affect clock entrainment to produce a circadian day and night while preserving the ability of the staff to provide care. [95]. This resulted in enhanced day-night activity rhythms and increased weight gain critical in premature infants. These initial cohorts of infants are being tracked in a longitudinal study to ascertain if there are lifetime benefits of maintaining circadian cohesion during development.

## 5. Conclusion

Our understanding of ourselves as photoperiodic organisms has given new importance to research into the neural basis for photoperiodic responses. While much progress has been made in demonstrating the ability of the mammalian master circadian clock, the SCN, to encode photoperiodic information, as well as in identifying various brain structures involved in many types of photoperiodic outputs observed, there remains a great deal to be understood. At present, there is a myriad of day-length-dependent responses in physiology that range from purely light driven, to transiently sustained, to permanent. More must be learned about the physiology that underlies the differences between these types of responses. Some may rely on the SCN, while others may act independently of the clock. Likewise, many clearly depend on melatonin but others may not require melatonin signaling to occur. We have already observed the benefits that medicine and society may see when photoperiodic research is applied, but to truly modify or medically intervene with this system, a better understanding is required.
